# TAD-SIE: sample size estimation for clinical randomized controlled trials using a Trend-Adaptive Design with a Synthetic-Intervention-Based Estimator

**DOI:** 10.1186/s13063-024-08661-1

**Published:** 2025-01-29

**Authors:** Sayeri Lala, Niraj K. Jha

**Affiliations:** Department of Electrical and Computer Engineering, Princeton University, Princeton, 08544 NJ USA

**Keywords:** Adaptive design, Clinical randomized controlled trials, Counterfactual estimation, Crossover design, Sample size estimation, Synthetic intervention

## Abstract

**Background:**

Phase-3 clinical trials provide the highest level of evidence on drug safety and effectiveness needed for market approval by implementing large randomized controlled trials (RCTs). However, 30–40% of these trials fail mainly because such studies have inadequate sample sizes, stemming from the inability to obtain accurate initial estimates of average treatment effect parameters.

**Methods:**

To remove this obstacle from the drug development cycle, we present a new algorithm called Trend-Adaptive Design with a Synthetic-Intervention-Based Estimator (TAD-SIE) that powers a parallel-group trial, a standard RCT design, by leveraging a state-of-the-art hypothesis testing strategy and a novel trend-adaptive design (TAD). Specifically, TAD-SIE uses synthetic intervention (SI) to estimate individual treatment effects and thereby simulate a cross-over design, which makes it easier for a trial to reach target power within trial constraints (e.g., sample size limits). To estimate sample sizes, TAD-SIE implements a new TAD tailored to SI given that using it violates assumptions under standard TADs. In addition, our TAD overcomes the ineffectiveness of standard TADs by allowing sample sizes to be increased across iterations without any condition while controlling significance level with futility stopping. Our TAD also introduces a hyperparameter that enables trial designers to trade off between accuracy and efficiency (sample size and number of iterations) of the solution.

**Results:**

On a real-world Phase-3 clinical RCT (i.e., a two-arm parallel-group superiority trial with an equal number of subjects per arm), TAD-SIE obtains operating points ranging between 63% to 84% power and 3% to 6% significance level in contrast to baseline algorithms that get at best 49% power and 6% significance level.

**Conclusion:**

TAD-SIE is a superior TAD that can be used to reach typical target operating points but only for trials with rapidly measurable primary outcomes due to its sequential nature. The framework is useful to practitioners interested in leveraging the SI algorithm for their study design.

## Introduction

The randomized controlled trial (RCT) is the gold-standard approach for establishing treatment effects in phase-3 trials, which in practice requires hundreds to thousands of subjects [1, 2]. Despite phase-3 trials accounting for 60% of R&D investment for clinical trials (approximately $500 million USD per drug in year 2019), 30–40% of these trials fail to proceed to market approval [3, 4], primarily because they have insufficient sample size [5]. This is because initial sample size calculations are based on noisy estimates of treatment effect parameters obtained from prior studies, if available, or internal pilot studies [1, 6].

Adaptive trial designs have been developed to improve sample size estimates by adjusting them based on interim analyses conducted over the course of the trial. Standard approaches for doing so include group sequential designs (GSD), stochastic curtailment, and trend-adaptive designs (TAD). A GSD can decrease the initial planned sample size by testing at each interim analysis. In order to control the significance level, the test boundary used at each analysis needs to be increased [1]. Consequently, a GSD yields marginal reductions in sample sizes when the standardized treatment effect is larger than that used for planning [7]. For GSDs to be useful, a trial designer still needs to have a good prior over the range of the standardized treatment effect, otherwise risks underpowering or overpowering the study [7]. Stochastic curtailment is another approach that can decrease the initial planned sample size by terminating trials that appear futile [1, 8]. It determines futility based on conditional power (CP), which extrapolates power at the final sample size conditioned on the value of the interim test statistic. If CP at any interim analysis lies below some pre-specified futility threshold, the trial is terminated. Since stochastic curtailment is used to control the significance level and reduce resources expended by terminating early [8], it cannot be used to appropriately power studies. Instead of decreasing the initial planned sample size, TADs can increase it based on trends observed from interim data. Among trend-adaptive algorithms, those based on CP have been recommended since they can control significance level without making statistical adjustments to the test statistic and test critical value [1, 7, 9]. They do this by only permitting sample size increases when the trend in the data appears “promising,” a condition determined by CP at interim analysis. If CP is sufficiently high, i.e., 50% [9], or lies within a promising range [7], the final sample size can be increased. In practice, such TADs have marginal impact on increasing power since the probability of satisfying the CP criterion at an interim analysis remains low [7].

Given the limitations of existing adaptive designs, we present a new solution called Trend-Adaptive Design with a Synthetic-Intervention-Based Estimator (TAD-SIE). In contrast to existing TADs, it repeatedly increases the sample size based on *individual* treatment effect (ITE) estimates obtained under synthetic intervention (SI) [10] and controls for significance level with futility stopping. Leveraging ITEs increases power and permitting sample size increases, while stopping for futility, enables TAD-SIE to yield solutions with better power and significance level compared to existing TADs. TAD-SIE also introduces a hyperparameter that allows users to toggle between solutions that are either more sample- or time-efficient. We empirically demonstrate TAD-SIE’s efficacy over baseline strategies on a sample real-world RCT dataset. Our work is useful for practitioners interested in learning how to integrate SI into TADs.

The rest of the article is organized as follows. We provide background on topics relevant to our framework in the “Background” section and then present the framework in the “Methodology” section. We explain how we evaluate performance in the “Performance evaluation” section. We present our results in the “Results” section, discuss their implications in the “Discussion” section, and draw conclusions in the “Conclusion” section.

## Background

In this section, we provide background on concepts relevant to understanding TAD-SIE, which include the SI algorithm and a hypothesis testing procedure based on the SI algorithm.

### SI

SI [10] is an algorithm that estimates counterfactual outcome trajectories under interventions different from the one that a unit (e.g., a patient) was exposed to during the intervention period. To do this, first, it assumes that a pool of donor units exposed to the interventions (including the control) exists and that each unit has been assigned to the control arm during the pre-intervention period. Then, for a given target unit observed under some intervention *j*, SI calculates its counterfactual outcome under a different intervention *k* by weighting the post-intervention outcomes across the donor units exposed to intervention *k*. It uses principal component regression to learn weights over the donor units such that the weighted sum of their pre-intervention outcomes best predicts the target unit’s pre-intervention outcome.

### Hypothesis testing with SI

**Fig. 1:**
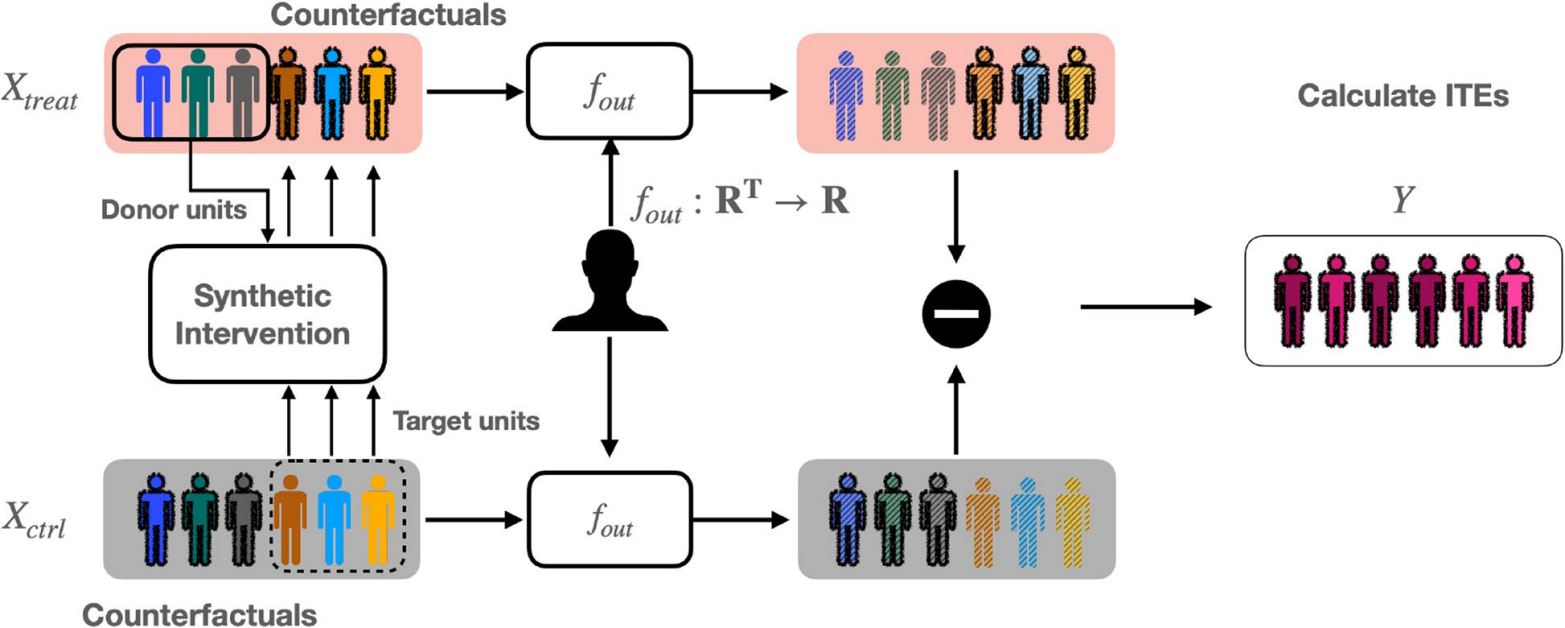
The flowchart of the ITE estimation step in SECRETS for a parallel two-arm design. Counterfactual treatment outcomes for each participant in the control group are generated by using the treatment arm as the donor data; counterfactual control outcomes for participants in the treatment arm are generated analogously (not shown for visual simplicity). After applying SI, SECRETS transforms each time-series datum to a scalar by applying the outcome function defined by the trial investigator. SECRETS then calculates the ITEs by taking the pairwise difference between each patient’s outcome under the treatment and control conditions

**Fig. 2:**
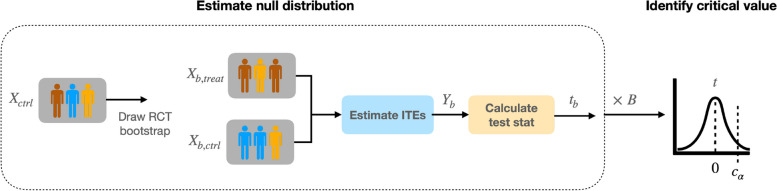
The flowchart of the hypothesis testing step in SECRETS for a parallel two-arm design. SECRETS first estimates the null distribution with bootstrap sampling and then uses it to tune the testing critical value

Previously, we developed a framework called SECRETS (Subject-Efficient Clinical Randomized Controlled Trials using Synthetic Intervention) [11] that applies the SI algorithm to increase the power of an already-conducted clinical RCT. SECRETS first estimates the ITE per participant using SI to reduce intersubject variability [1], as shown in Fig. 1. Afterwards, it uses a novel bootstrapping strategy illustrated in Fig. 2 to implement a hypothesis test that appropriately controls the type-1 error rate, given the dependencies present among the estimated ITEs.

## Methodology

**Fig. 3:**
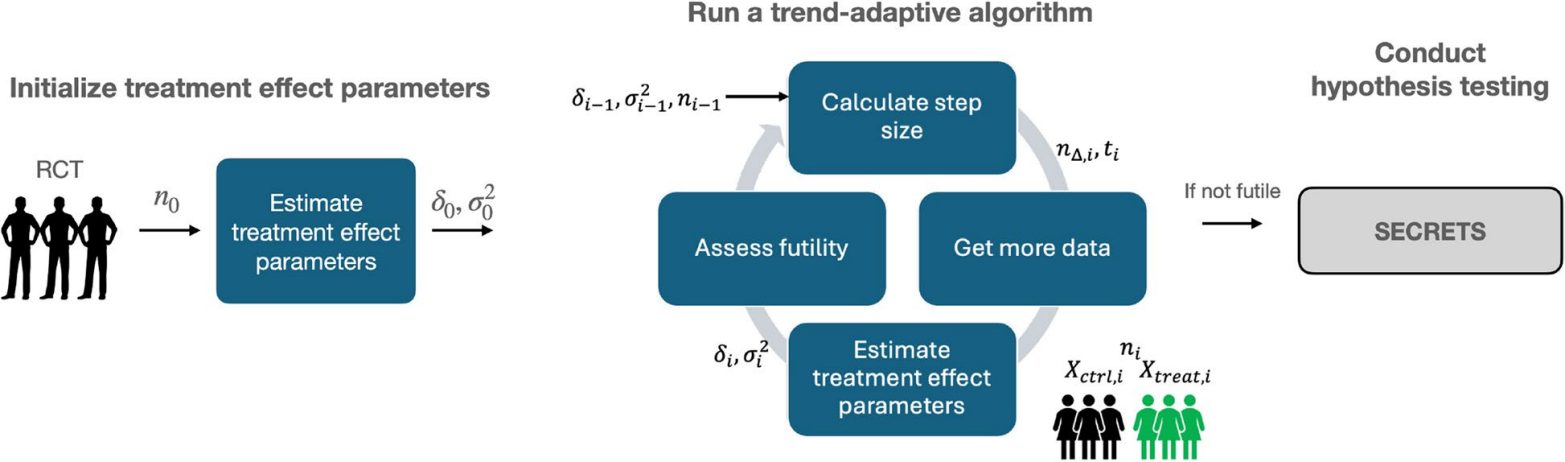
The flowchart of the TAD-SIE framework

TAD-SIE is a new TAD that leverages the SI-based hypothesis testing framework, i.e., SECRETS, to yield solutions converging closer to the target power and significance level. The framework’s flowchart is illustrated in Fig. 3 and is shown for a common design, i.e., the two-arm parallel superiority trial with an equal number of participants per arm [1]. First, it implements an internal pilot study of size $n_0$ to obtain initial estimates over key treatment effect parameters including the average treatment effect (ATE) ($\delta _0$) and variance ($\sigma _0^2$) under SECRETS. Then, TAD-SIE implements a trend-adaptive algorithm that iteratively refines the estimates to converge to an accurate estimate of the target sample size needed for target power. Specifically, TAD-SIE determines how much to increase the current sample size by ($n_{\Delta ,i}$) based on user-specified parameters and prior estimates of treatment effect parameters obtained from the pilot study or prior iteration. It collects the additional RCT data, updates the estimates of treatment effect parameters using the accrued RCT data ($X_{ctrl,i}, \ X_{treat,i}$), and then assesses futility based on quantities computed in the iteration, including information fraction $t_i$, which estimates what fraction of the final sample size has been collected. Finally, if the trial is not futile, TAD-SIE runs SECRETS to perform hypothesis testing.

Next, we describe the procedures underlying TAD-SIE in more detail.

### Estimation of treatment effect parameters

TAD-SIE estimates the mean and the variance of the ITEs using Algorithm 1. The ATE is given by the average of the ITEs calculated under SECRETS (line 6). Calculating the variance requires a new procedure because the standard sample size formula requires that the ITEs be independently and identically distributed (i.i.d.) according to a Gaussian [12]. Since the distribution of the ATE under SECRETS is approximated by a normal distribution, per the theorem on Dependency Neighborhoods based on Stein’s method [13], the procedure first estimates the variance of the ATE and uses it to estimate the variance of a set of hypothetical ITEs satisfying the i.i.d. assumption that would also yield the observed distribution of the ATE. Specifically, it estimates the variance of the ATE under SECRETS with bootstrap sampling (lines 7–12) and then calculates the final variance based on the relationship between variance of a mean and variance of underlying i.i.d. samples (line 13).

**Figure:**
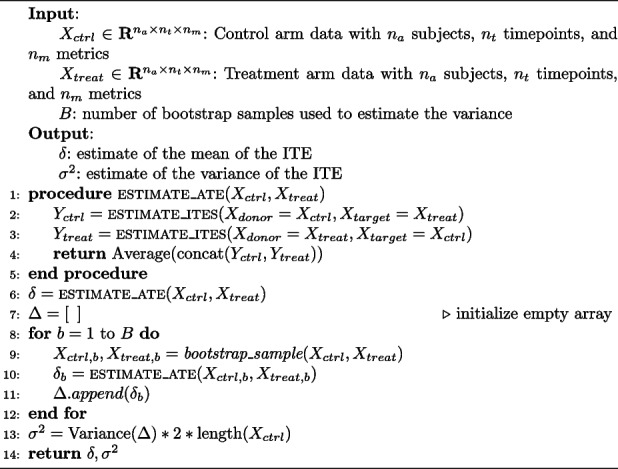
**Algorithm 1** *estimate_moments*

### Step size calculation

TAD-SIE determines by how much to increase the current sample size using Algorithm 2. First, it estimates the arm size needed for target performance using the sample size formula evaluated under the target performance level (significance level $\alpha$ and power $1-\beta$) and current estimates of the treatment effect parameters (line 1). Then, it calculates the step size $n_{step}$ based on user-specified hyperparameters. Specifically, it scales the target sample size increase according to the *step_size_scale_factor* (capped to 1 to avoid overpowering) and ensures that the step size is nonnegative and does not exceed resource constraints determined by the maximum arm size $n_{max}$ (lines 2–3). *step_size_scale_factor* determines how fast the algorithm terminates, with larger values resulting in fewer iterations at the cost of larger sample sizes since the sample size is increased at a higher rate. In addition, Algorithm 2 also estimates the information fraction *t* resulting from the sample size increase since this is used for futility stopping. *t* is calculated by determining the maximum possible step size and then taking the ratio of the updated sample size over the estimated final sample size (lines 4–6).

If $n_{step}$ is 0, TAD-SIE terminates the trend-adaptive algorithm. Otherwise, it collects the additional RCT data, uses the dataset to revise the treatment effect parameters (Algorithm 1), and then checks for futility.

**Figure:**
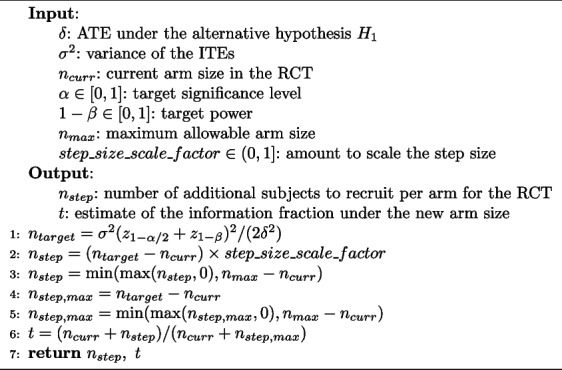
**Algorithm 2** *get_step_size*

### Futility assessment

TAD-SIE checks for futility by implementing the stochastic curtailment procedure given in Algorithm 3. It calculates CP based on estimates of treatment effect parameters, the current sample size, and information fraction (lines 1–2), and then marks the trial as futile if the CP estimate is below *futility_power_boundary*, a user-specified hyperparameter (line 3). TAD-SIE terminates the trend-adaptive algorithm if the trial is futile, in which case it fails to reject the null hypothesis and accepts it by convention [12]. If the trial does not fail by futility, TAD-SIE performs hypothesis testing with SECRETS using the final RCT dataset.

**Figure:**
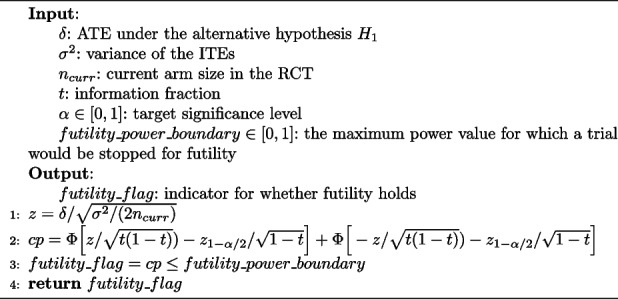
**Algorithm 3** *check_for_futility*

## Performance evaluation

In this section, we describe performance metrics and the dataset used to evaluate TAD-SIE. We describe the baseline algorithms against which we compare our approach and describe the ablation studies done to demonstrate the contribution of each novel component of TAD-SIE. We also provide implementation details of the algorithms and experiments.

### Performance metrics

We set target power, $1-\beta _{target}$, to 80% and target significance level, $\alpha _{target}$, to 5%, following typical target operating points [1]. We measure power and significance level obtained by TAD-SIE and baseline algorithms following the approach from [14], which simulates many trials under the alternative and null settings and calculates the percentage of trials where the test procedure returns a reject, respectively. Specifically, we simulate a trial under the alternative setting by constructing new control and treatment arms with subjects sampled with replacement from the original RCT’s control and treatment arms, respectively. Similarly, we simulate the null setting by constructing both the control and treatment arms with subjects sampled with replacement from the original RCT’s control arm.

For TAD-SIE, we also report the final arm size and number of iterations that a trial takes in order to characterize TAD-SIE’s efficiency.

### Dataset

We evaluate the framework on a real-world clinical phase-3 parallel-group RCT and demonstrate it for a two-arm superiority trial, a design typically adopted in clinical RCTs [1]. We obtained the dataset for a sample trial, e.g., CHAMP (NCT01581281), [15, 16], from the National Institute of Neurologic Disease and Stroke (NINDS) [17]. Additional details can be found in the Appendix: Dataset section.

### Baselines

We compare TAD-SIE against two baseline algorithms. Both algorithms implement parallel-group RCTs following a two-arm superiority setup and therefore use the two-sample *t*-test for independent samples with unequal variances for hypothesis testing [12]. The approaches differ in how they determine the final sample size.

The fixed sample design baseline is a standard approach for study planning that calculates the sample size required for target power and target significance level using Eq. (1), where the ATE $\delta$ and variances for the control and treatment arms, $\sigma _{ctrl}^2$ and $\sigma _{treat}^2$, are pre-specified or estimated from a prior study [1, 7]. Since domain knowledge may not be available to appropriately pre-specify these parameters, the baseline implements a small internal pilot study to estimate these parameters [6]. The baseline then conducts an RCT according to the calculated sample size, which is capped at a maximum arm size set by trial constraints [7].

1 $$\begin{aligned} n_{a}=\frac{\left( \sigma _{control}^{2}+\sigma _{treat}^{2}\right) \left( z_{1-\alpha /2}+z_{1-\beta }\right) ^{2}}{\delta ^{2}} \end{aligned}$$

We also implement a traditional adaptive design that can increase the initial sample size calculated by the fixed sample design strategy in order to increase power. We adopt a TAD based on CP and specifically implement the algorithm from [9], given its simplicity, which we refer to as standard TAD. Specifically, at each interim analysis, it increases the sample size according to the sample size formula when CP is above 50%.

### Ablation studies

We validate the importance of each component in TAD-SIE using several ablation studies. First, we swap the proposed variance estimation procedure (Algorithm 1) with a naive approach that uses the variance of the ITEs (thereby assuming that the ITEs are i.i.d. [12]). Next, we swap the proposed TAD with a standard TAD [9] to show that an approach implementing a rule for sample size increases, based on control over significance level, will fail to reach the target operating point since increases are rare. Finally, we modify TAD-SIE so that it performs sample size estimation based on standard hypothesis testing instead of SECRETS to show that a TAD designed for a powerful testing scheme is necessary for reaching the target operating point. Additional implementation details for the ablations are presented in the Appendix: Ablations section.

### Implementation details

We describe the hyperparameters used by TAD-SIE and the baseline algorithms. Additional experimental and computing details are provided in the Appendix: Implementations details section.

Hyperparameters for TAD-SIE are reported in Table 1. While most hyperparameters can be determined from prior work, *step_size_scale_factor* is a new hyperparameter introduced by TAD-SIE; hence, we sweep over values over the domain of the hyperparameter in increments of 0.1 to characterize its effect on performance. Hyperparameter details for SECRETS are specified in the Appendix: Implementation details section.

**Table 1:** Hyperparameters used for TAD-SIE. The "Reference" column lists references that support the choice of the hyperparameter value

Hyperparameter	Value	Reference
$n_{0}$	30	[18, 19]
*B*	100	[11]
$\alpha$	5%	[1]
$1-\beta$	80%	[1]
$n_{max}$	1500	[20]
*step_size_scale_factor*	(0, 1]	n/a
*futility_power_boundary*	$[10,20\%]$	[21]

The baseline algorithms use the same values used by TAD-SIE for $n_{0}$, $\alpha$, $1-\beta$, and $n_{max}$. For Standard TAD, we set the number of interim analyses to 1 since this is common in practice [7] and perform interim analysis at the initial planned sample size by setting $t=0.99$ (CP is undefined at $t=1$) since this is ideal for assessing whether the sample size can be increased and the amount by which it needs to be increased [7].

## Results

First, we compare the performance of TAD-SIE against baseline strategies. Then, we analyze the effect of hyperparameters on TAD-SIE’s performance. Finally, we analyze the results from the ablation studies.

### TAD-SIE vs. baselines

**Fig. 4:**
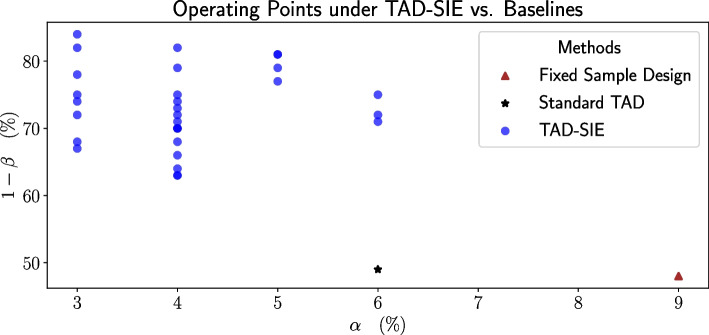
A comparison of the operating points defined by significance level and power obtained across the different methods

TAD-SIE yields superior operating points compared to the baseline strategies, as shown in Fig. 4. Fixed sample design results in lower power (48%) and higher significance level (9%) since it uses noisy estimates obtained from a small pilot study to estimate the required sample size. Standard TAD improves upon the fixed sample design by allowing the initial sample size estimated under fixed sample design to be increased based on interim data; however, the actual improvement is marginal (i.e., power increases to 49% while significance level decreases to 6%) since few trials perform an increase when $H_1$ holds given that Standard TAD imposes a stringent condition for when the sample size can be increased in order to control the significance level. Specifically, when $H_0$ holds, 7% of trials meet the criterion and 4% of trials increase the sample size, thereby preventing type-1 inflation; however, when $H_1$ holds, only 51% of trials meet the criterion and 17% of trials increase the sample size, thereby precluding gains in power. In contrast, the operating points generated with TAD-SIE under different combinations of *step_size_scale_factor* and *futility_power_boundary* have substantially better performance, with power ranging between 63% to 84% and significance levels ranging between 3 and 6% (90% of hyperparameter configurations have significance levels no worse than 5%).

### Effect of hyperparameters on TAD-SIE’s performance

**Fig. 5:**
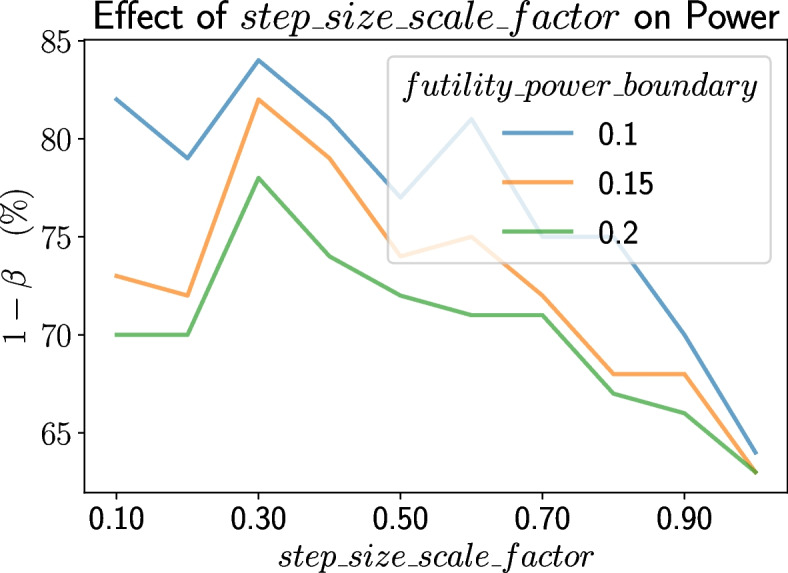
The effect of *step_size_scale_factor* and *futility_power_boundary* on power under TAD-SIE

**Fig. 6:**
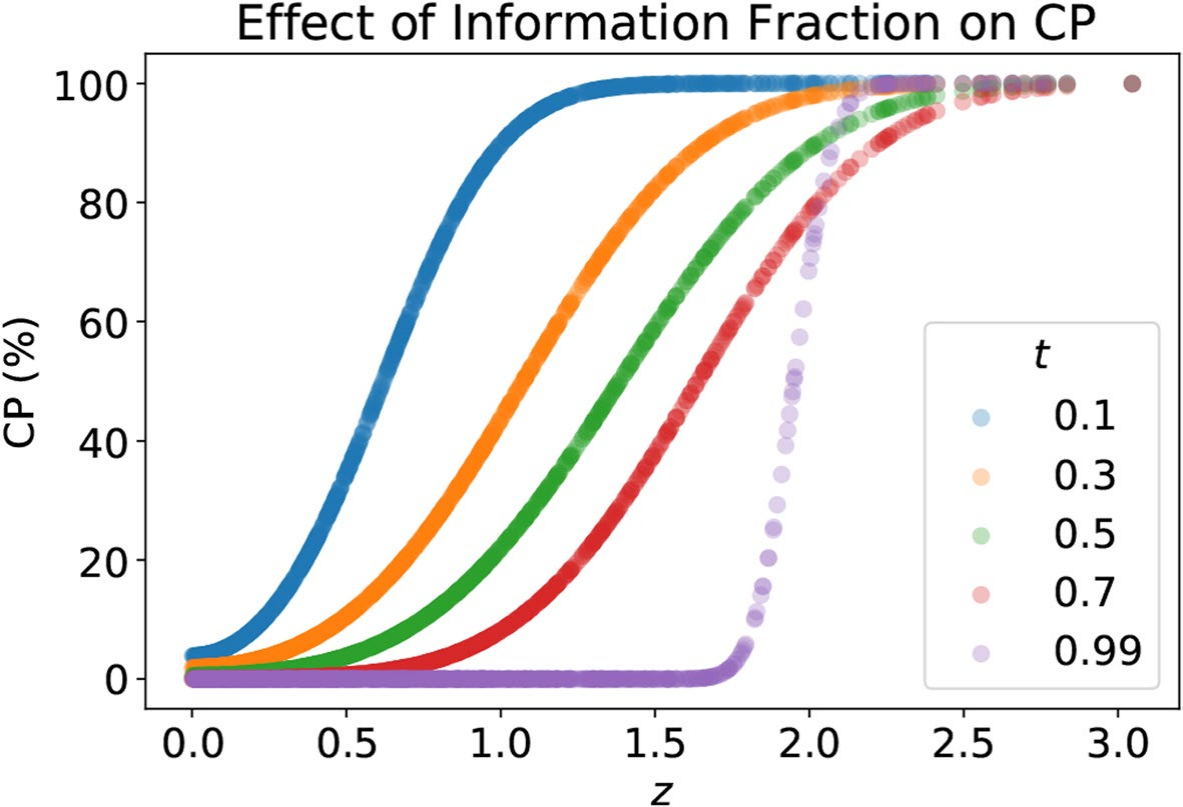
CP under two-sided testing as a function of *z*, the interim test statistic, and *t*, the information fraction. Function plotted over nonnegative values of *z* since the function is symmetric in *z*

Since TAD-SIE yields operating points spanning a large range over power, we characterize the effect of the *step_size_scale_factor* on power, which is shown in Fig. 5. For a given value of *futility_power_boundary*, increasing *step_size_scale_factor* generally decreases power because it increases the chance for futility stopping. Specifically, increasing *step_size_scale_factor* increases the information fraction *t* since *t* is more likely to be determined by *step_size_scale_factor* at initial interim analyses when the arm size is small (per line 6 in Algorithm 2). A higher information fraction shrinks CP over a large range of test statistic values, as shown in Fig. 6, thereby triggering futility stopping. Note that increasing *futility_power_boundary* decreases power across all values of *step_size_scale_factor* by invoking futility stopping more readily.

**Fig. 7:**
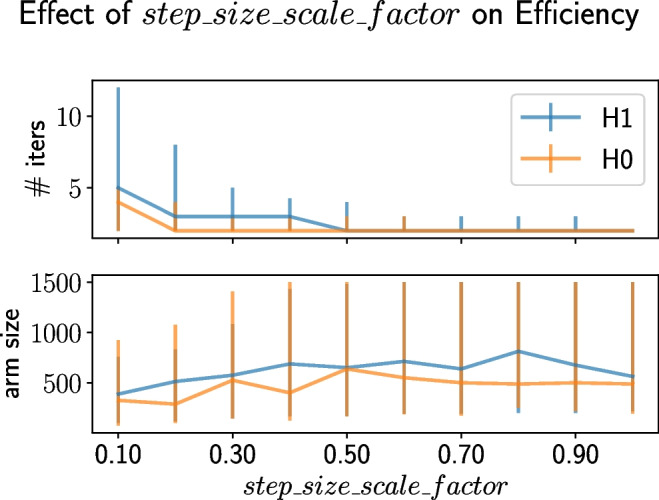
The effect of *step_size_scale_factor* on the number of iterations incurred and arm size obtained under TAD-SIE when the futility power boundary is 0.10. The curves represent the medians and the error bars correspond to interquartile ranges

In addition to its impact on power, *step_size_scale_factor* affects the solution’s efficiency (i.e., final sample size and number of iterations). The effect is shown in Fig. 7. Increasing *step_size_scale_factor* improves the time efficiency but reduces the sample efficiency. For example, when *step_size_scale_factor* is 0.1, the median number of iterations incurred is 5 and the median arm size is 387.5, and when *step_size_scale_factor* is 0.9, the median number of iterations incurred is 2 and the median arm size is 562.5. This tradeoff occurs because increasing *step_size_scale_factor* increases initial step sizes, which can overshoot and cause the algorithm to terminate earlier since the step size goes to zero in a later iteration; in addition, increasing *step_size_scale_factor* increases the chance for futility stopping through its effect on information fraction. Similar trends are observed when $H_0$ holds although the tradeoff is less pronounced since futility stopping is more likely to be invoked.

### Ablation studies

**Fig. 8:**
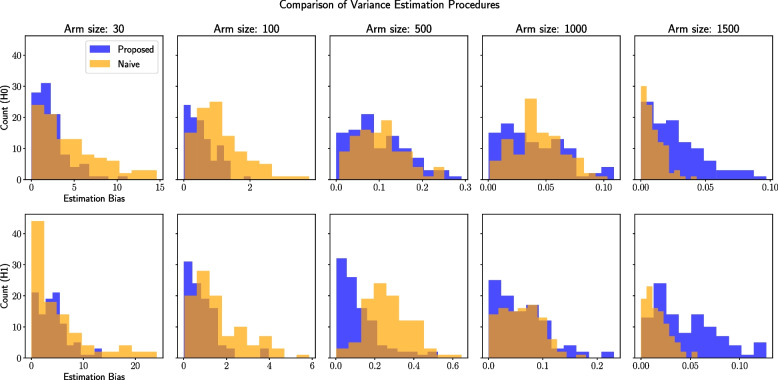
Comparison of the magnitude of the estimation bias of the variance of the ATE under the proposed and naive methods across different arm sizes (columns) and settings, i.e., $H_1$ or $H_0$ (rows). Histogram counts per arm size are obtained from 100 bootstrap samples

Next, we present results from the ablation studies. First, we show that the proposed variance estimation procedure used in Algorithm 1 is more effective than the naive variance estimation strategy. As can be seen from Fig. 8, the proposed strategy generally has lower bias in the estimation of the variance of the ATE compared to the naive strategy, especially under low sample sizes (at the maximum sample size, the range of the estimation bias is larger under the proposed strategy but the range is small). This result demonstrates that our variance estimation procedure is more effective by accounting for the dependencies present across the ITEs compared to the naive approach that assumes that the ITEs are i.i.d.

**Fig. 9:**
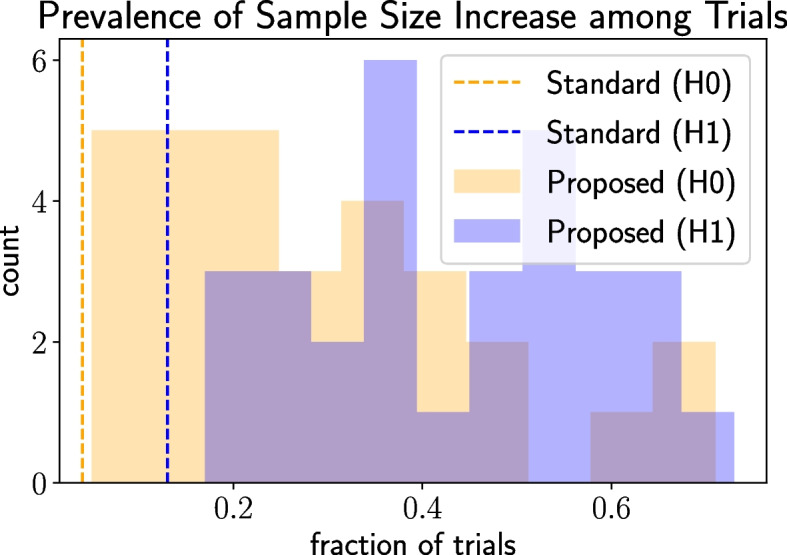
Comparison of the fraction of trials that increase the sample size at least once under the standard and proposed TADs. The histogram over the proposed version is generated by sweeping over hyperparameter configurations

Next, we demonstrate that the proposed trend-adaptive algorithm is more effective than the standard one. Swapping the proposed algorithm with the standard one yields a single operating point of 62% power and 6% significance level (the standard trend-adaptive design does not introduce the *step_size_scale_factor* and *futility_power_boundary* hyperparameters). This operating point is inferior to the range of operating points generated under the proposed TAD. Power drops substantially under the standard algorithm because it imposes a stringent condition based on CP that precludes trials from increasing their sample sizes after the initial sample size calculation in order to control the significance level. The effect is seen in Fig. 9. In particular, when $H_1$ holds, only 13% of trials increase the sample size at least once under the standard algorithm while 17% to 73% do so under the proposed algorithm. Similar trends are observed under $H_0$ although the fraction of trials performing an increase is lower due to futility stopping.

**Fig. 10:**
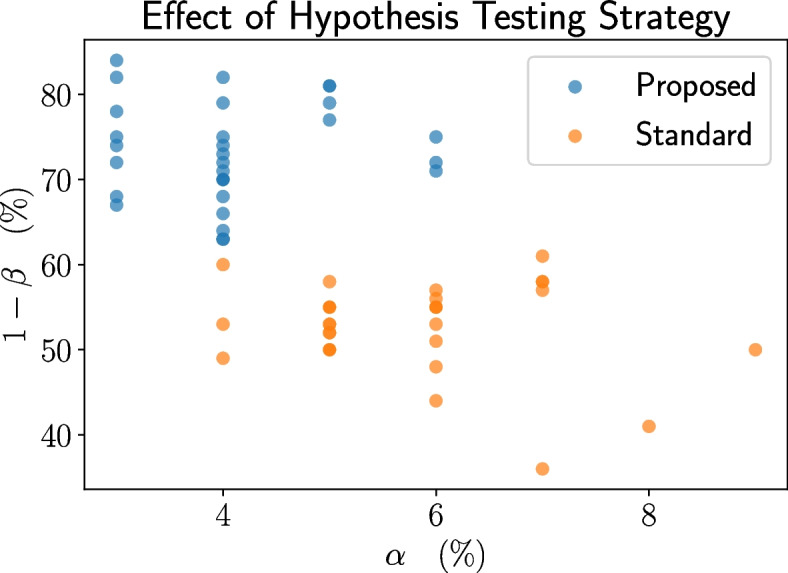
Comparison of operating points generated under the proposed hypothesis testing scheme compared to the standard one

Finally, we demonstrate that the proposed hypothesis testing scheme is more effective than the standard one. Figure 10 shows that the algorithm that uses SECRETS for hypothesis testing yields superior operating points compared to the version that uses the standard two-sample *t*-test; for example, the proposed strategy gets at least 77% power and 5% significance level while the standard strategy gets at best 58% power and 5% significance level. SECRETS is more effective at reaching target power because it simulates the cross-over design to boost power.

## Discussion

Having demonstrated the superiority of TAD-SIE over existing frameworks for sample size estimation and having vetted the significance of each component to TAD-SIE’s performance through ablation studies, we discuss practical issues, i.e., design decisions and limitations, to guide the practitioner in applying the TAD-SIE framework. Specifically, TAD-SIE introduces the *step_size_scale_factor* hyperparameter that controls the rate at which the sample size is increased. While this hyperparameter can be selected based solely on resource constraints, it also affects the operating points obtained, with larger values yielding lower power through its interaction with futility stopping. Our empirical findings suggest that moderate values (0.3–0.6) can balance the two objectives, that is, achieve operating points with high power while maintaining some efficiency in the trial duration and sample size. However, TAD-SIE inherits limitations of adaptive designs, namely that the iterative nature of the algorithm prevents it from being practical in trials conducted on outcomes that take too long to measure (e.g., mortality). Our future work will extend TAD-SIE to address such settings by having it estimate the primary outcome from rapidly measurable surrogate outcomes rather than directly measuring its value [22], given that trials collect data across other response variables [1, 23].

## Conclusion

In conclusion, we presented TAD-SIE, a novel TAD that integrates SI to better reach target power and significance level for a parallel-group RCT in the absence of reliable sample size estimates obtained for study planning. Specifically, TAD-SIE uses estimates of ITEs obtained under SI to increase power and introduces a procedure to effectively calculate the parameters defining the treatment effect given that SI induces dependencies across the ITEs. Furthermore, in contrast to a standard TAD, TAD-SIE permits many iterations of sample size increases while controlling significance level with futility stopping. We have demonstrated TAD-SIE’s effectiveness over baseline approaches on a real-world phase-3 clinical RCT, showing that it obtains superior operating points ranging between 63% to 84% power and 3% to 6% significance level, in contrast to baseline methods that get at best 49% power and 6% significance level. We have also characterized the effect of a new hyperparameter introduced in TAD-SIE that trades off between accuracy and efficiency (e.g., time and sample size) of the solution in order to guide the practitioner. To make TAD-SIE broadly applicable, our future work will speed up each iteration of the algorithm by predicting the outcome of interest based on rapidly measurable surrogate outcomes.

## Data Availability

The RCT dataset used for evaluation can be obtained from NINDS [17] by filling out the following form (https://www.ninds.nih.gov/sites/default/files/migrate-documents/sig_form_revised_508c.pdf) and emailing it to NINDS (CRLiaison@ninds.nih.gov). Under the “Dataset Being Requested” section, set the Trial acronym to “CHAMP”, NCT# to 01581281, Trial Title to “The Childhood and Adolescent Migraine Prevention Study (CHAMP)”, and Trial PI Name to “Scott W. Powers, PhD”. Flowcharts and pseudocodes for algorithms have been provided in the Methodology section.

## Appendix A: Performance evaluation

In this section, we present additional details on our experimental setup.

### Dataset

The CHAMP study [15] conducted an RCT to compare the effect of different medications (amitriptyline and topiramate) on mitigating headaches. We construct a dataset corresponding to a two-arm superiority trial. Specifically, we set the control arm to be the group exposed to amitriptyline and the treatment arm to be the group exposed to topiramate and define the ATE to be the difference between their average outcomes, where the outcome is the change in the score on the Pediatric Migraine Disability Assessment Scale between the 24-week endpoint and the baseline visit. This setup yields a dataset of 204 subjects, with 106 in the amitriptyline group and 98 in the topiramate group, where the ATE is − 3.17 units, somewhat comparable to the − 4.3 units reported in the study [16].

### Implementation details

For SECRETS, we use the hyperparameter settings from [11].

For evaluation, we set the number of trials to 100 since this was sufficient for powers and significance levels to stabilize. For implementation ease, we pre-computed results across arm sizes sampled between the pilot study size to the maximum arm size and then projected interim and final sample sizes to this set. We sampled in increments of 25 since this was sufficient for consecutively sampled arm sizes to have comparable means and variances.

We implemented the framework and experiments with Python using standard numerical packages and conducted experiments using 28–32 CPU cores, 2–4GB of memory per CPU, and Intel processors (e.g., 2.4 GHz Skylake and 2.6 GHz Intel Skylake).

### Ablations

To implement the ablation on the hypothesis testing scheme, we appropriately modify TAD-SIE to make it suitable for standard hypothesis testing. Specifically, we modify *estimate_moments* to calculate the ATE and variances of the outcome over the control and treatment groups based on the original RCT data; these equations are captured in Eq. (2), where $o_{control}$ and $o_{treat}$ are vectors that contain primary outcomes per subject in the control and treatment groups, respectively. We also modify *get_step_size* so that it takes in the variance for the control and treatment groups and uses the appropriate sample size formula, i.e., Eq. (1), in line 1 of Algorithm 2; we do not divide by two since Eq. (1) returns the sample size per arm. We modify *check_for_futility* similarly so that it takes in the two variance terms and uses the appropriate test statistic formula, i.e., Eq. (3) [12], in line 1 of Algorithm 2. Finally, we switch the hypothesis testing procedure from SECRETS to the two-sample *t*-test for independent samples with unequal variances [12].

2 $$\begin{aligned} \delta & =\text {Mean}(o_{treat})-\text {Mean}(o_{control}) \nonumber \\ \sigma _{ctrl}^2 & =\text {Var}(o_{control}) \nonumber \\ \sigma _{treat}^2 & =\text {Var}(o_{treat}) \end{aligned}$$

3 $$\begin{aligned} z=\frac{\delta }{\sqrt{(\sigma _{control}^2 + \sigma _{treat}^2)/n_a}} \end{aligned}$$

## Abbreviations

ATE: Average treatment effect
CP: Conditional power
GSD: Group sequential design
i.i.d.: Independent and identically distributed
ITE: Individual treatment effect
NINDS: National Institute of Neurologic Disease and Stroke
RCT: Randomized controlled trial
SECRETS: Subject-Efficient Clinical Randomized Controlled Trials using Synthetic Intervention
SI: Synthetic intervention
TAD: Trend-adaptive design
TAD-SIE: Trend-Adaptive Design with a Synthetic-Intervention-Based Estimator
